# Supplementary material for: MCARE enhances SERCA1 activity in fast-twitch muscle to maintain calcium handling and muscle integrity
Source: Nat Commun. 2025 Dec 10;17:629. doi: 10.1038/s41467-025-67358-4 (PMC12815910; doi:10.1038/s41467-025-67358-4)
Supplement: Supplementary file 1 — Supplementary Information [file 41467_2025_67358_MOESM1_ESM.pdf]

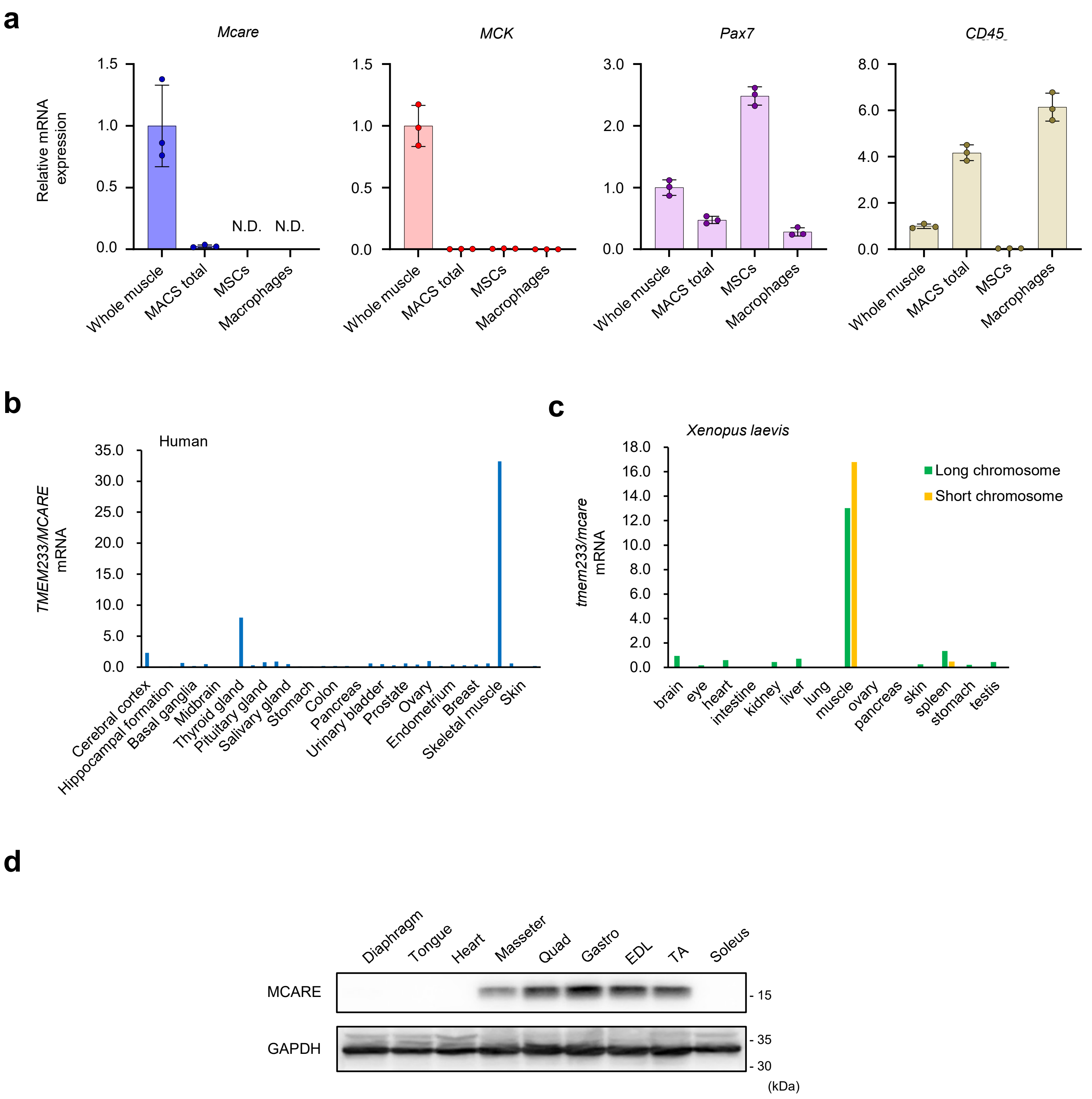

Supplementary Fig. 1 | The expression pattern of *Mcare* is conserved across vertebrate species and enriched in fast-twitch muscle fibers. a, Relative mRNA expression of *Mcare*, *MCK*, *Pax7,* and *CD45* in whole skeletal muscle tissue and total mononuclear cells (MACS total), muscle satellite cells (MSCs), and macrophages isolated by magnetic-activated cell sorting (MACS). n = 3 mice. Error bars represent mean ± SD. **b**, RNA-seq data from the GTEx project (http://commonfund.nih.gov/GTEx/) showing expression levels of *TMEM233/MCARE* across various human tissues. **c**, RNA-seq data from Xenbase (https://www.xenbase.org/xenbase/) showing expression of *tmem233/mcare* in various *Xenopus laevis* tissues. **d**, Western blot of adult mouse muscle tissues with MCARE- and GAPDH-specific antibodies.

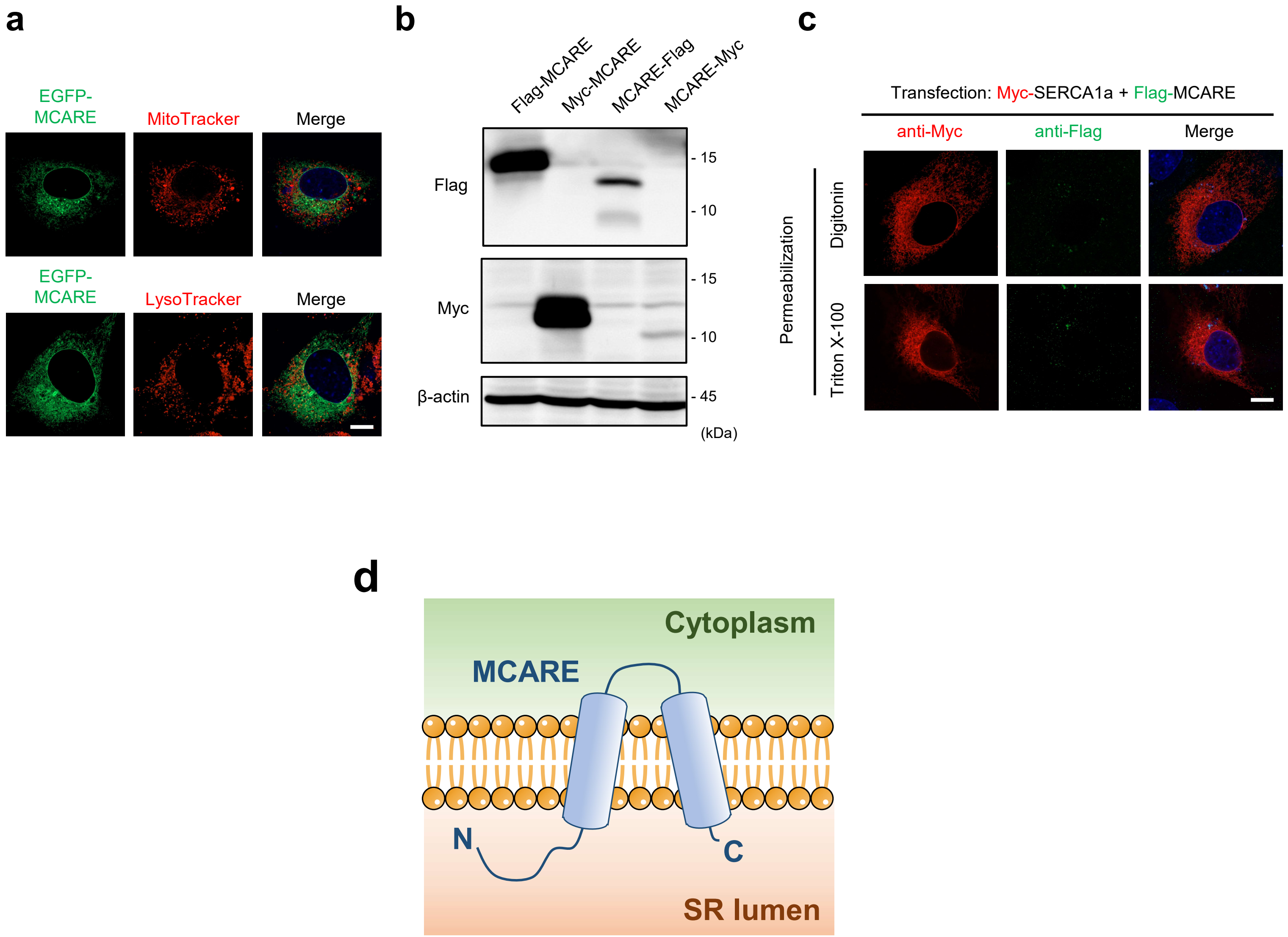

Supplementary Fig. 2 | Subcellular localization and predicted membrane topology of MCARE. a, Fluorescence microscopy images of C2C12 myoblasts expressing EGFP-tagged MCARE and co-stained with MitoTracker or LysoTracker. Scale bar, 10 μm. **b**, Western blot of HEK293T cells transfected with expression vectors encoding *Mcare* with N- or C-terminal Flag or Myc tags. **c**, Myc-SERCA1a and MCARE-Flag were transiently expressed in C2C12 myoblasts. Proteins were detected by immunofluorescence after permeabilization with Triton X-100 (complete) or Digitonin (selective). Scale bar, 10 μm. **d**, Schematic model of the predicted membrane topology of MCARE.

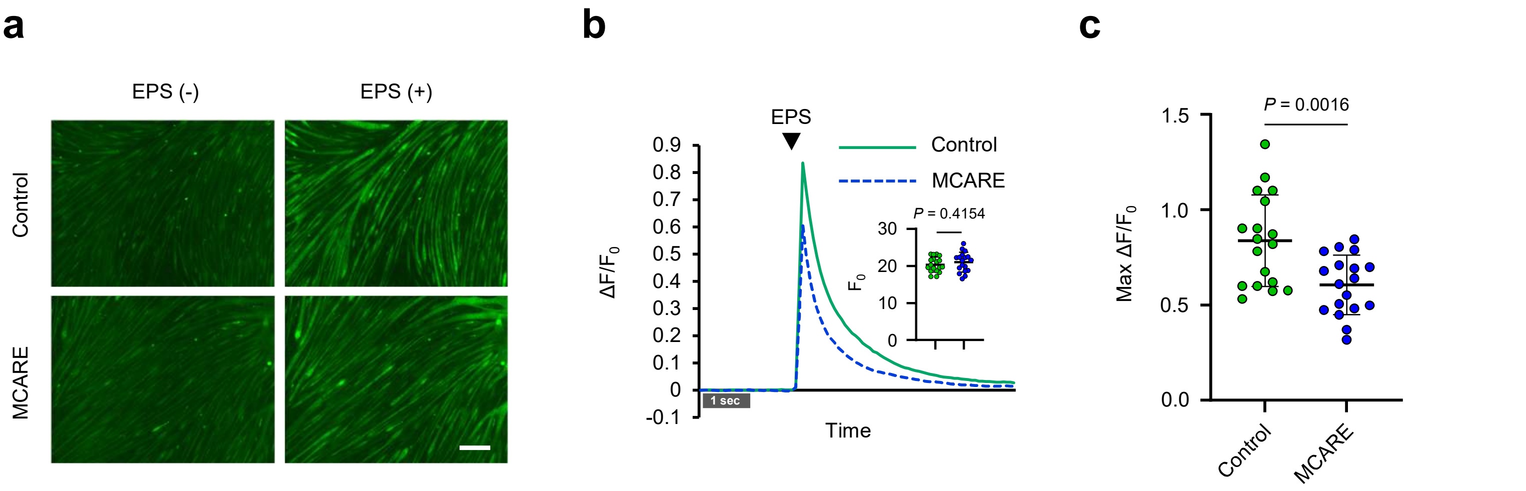

Supplementary Fig. 3 | Ca^{2+} responses under high-intensity EPS in C2C12 myotubes. a, Fluo-8 fluorescence images recorded in response to EPS (23 V, 6 ms) in C2C12 myotubes expressing LacZ (control) or MCARE. Scale bar, 200 µm. **b**, Changes in Ca^{2+} fluorescence intensity in response to EPS. Data include $\Delta F/F_0$ traces and F_0 from n = 18 myotubes. **c**, Maximum amplitude (Max $\Delta F/F_0$) of the EPS-induced Ca^{2+} fluorescence. n = 18 myotubes. Error bars represent mean ± SD. Statistical significance was assessed using a two-tailed unpaired Student's *t*-test.

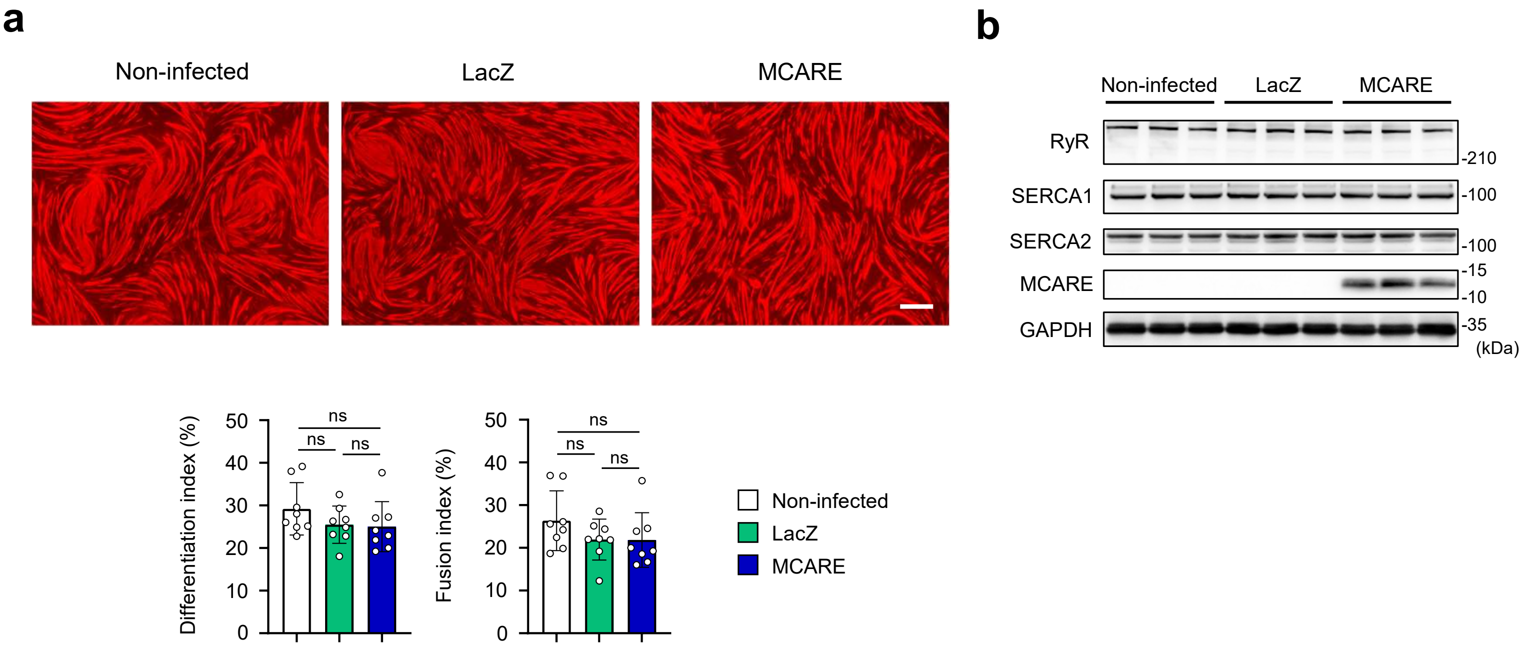

Supplementary Fig. 4 | MCARE does not alter myogenic differentiation or expression of RyR, SERCA1, or SERCA2. a, C2C12 myotubes were differentiated for 3 days, then either left uninfected or infected with adenovirus encoding *LacZ* or *Mcare*. After 2 additional days, cells were immunostained for MyHC, and the differentiation index (percentage of nuclei in MyHC-positive cells) and fusion index (percentage of MyHC-positive cells with three or more nuclei) were quantified. The upper panel shows fluorescence images of myotubes, and the lower panel shows the quantification results. Quantification was performed on eight randomly selected fields of view, each containing over 120 nuclei. Scale bar, 400 µm. Error bars represent mean ± SD. Statistical significance was assessed using two-tailed one-way ANOVA. ns, not significant. **b**, Western blot of non-infected C2C12 myotubes and LacZ- or MCARE-expressing C2C12 myotubes.

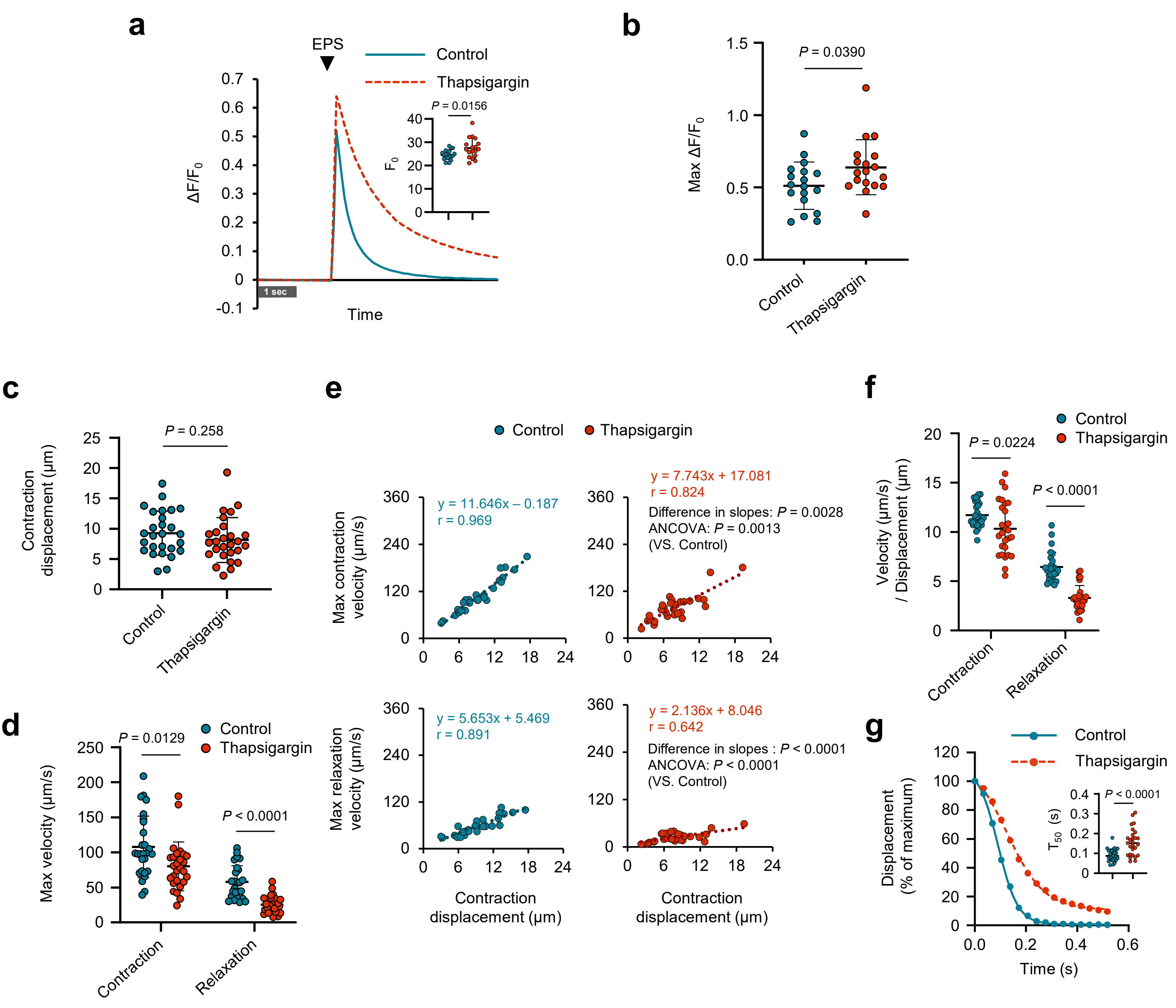

Supplementary Fig. 5 | Effects of thapsigargin on EPS-induced Ca^{2+} transients and contractile responses in C2C12 myotubes. a, Changes in Ca^{2+} fluorescence intensity in response to EPS (23 V, 2 ms). Data include $\Delta F/F_0$ traces and F_0 from n = 18 myotubes. **b**, Maximum $\Delta F/F_0$ of EPS-induced Ca^{2+} fluorescence. n = 18 myotubes. **c**, Contraction displacement of C2C12 myotubes treated with thapsigargin (100 nM, 30 min). n = 27 myotubes. **d**, Maximum contraction and relaxation velocities. n = 27 myotubes. **e**, Correlation analysis of contraction displacement with maximum contraction and relaxation velocities. Differences in regression slopes between control and thapsigargin groups were tested using two-tailed ANCOVA with contraction displacement as a covariate (contraction × displacement interaction: $F(1, 51) = 9.87$, $P = 0.0028$; relaxation × displacement interaction: $F(1, 51) = 20.96$, $P < 0.001$). The overall ANCOVA results for group effects are shown (contraction: $F(1, 51) = 11.61$, $P = 0.0013$; relaxation: $F(1, 51) = 72.14$, $P < 0.001$). n = 27 myotubes. **f**, Normalized contraction and relaxation velocities, adjusted by contraction displacement. n = 27 myotubes. **g**, Time-dependent changes in displacement from the peak contraction and T_{50} (half-relaxation time) after EPS. n = 27 myotubes. Error bars represent mean ± SD. Statistical significance was assessed using two-tailed unpaired Student's *t*-test (**a**, **b**, **c**, **d**, **f**, and **g**) or two-tailed ANCOVA (**e**).

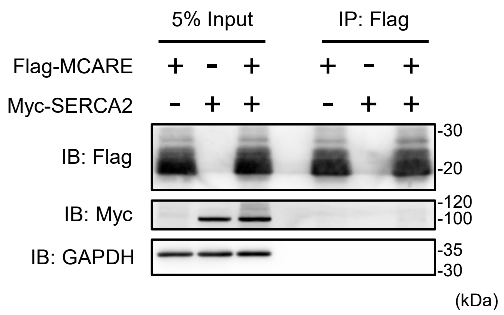

Supplementary Fig. 6 | Interaction between MCARE and SERCA2 in HEK293 cells. Western blot of IP Flag fractions and 5% input from HEK293 cells transfected with Flag-MCARE and Myc-SERCA2.

a

AGTGAAGTCTCATCTCCCTTGAGCCCGGAGCAGAAAGCTTTTCAGAGCCTCTGCAAGCCATGAGTCGACAG
CCAGAGCCCCAGGCTGCACGAACCGTGCGCTCCTCATCCCTCCCGGTGTCACCGACTTCGCCC**ATGTCTCA**
GTATGCTTCCCGCTCAGACTCAAAAGGAGCTTTGGACAGCAGCAGCCCGGAAGCCTATACAGAAGATGA
CAAGACCGAGGAGGACATACCTGCCCCCAGTAACTATCTATGGCTCACCATTATCTCGTGTTTCTGCCCAG
CGTACCCGGTCAACATCGTGGCTTTGGTCTTCTCCATCATG

gtgagttgggttgggaccaaaagcagctgcaaaggagagatgcgggggcggctctgcctctcatcgcccatgcttcttgcccaggacccatca
gtgctca ...... (Length, 31,467)

TCTCTGAACAGCTACAATGATGGAGACTACGAAGGAGCCAGGAGGCTGGGGCGGAACGCCAAGTGGGT
GGCCATTGCCTCCATCATCATCGGCCTGGTCATCATCGGCGTCTCCTGTGCGGTGCACTTCTCCAGGAA

gtaagtgggtgccccgaaaccctcctgctagtaagagcttttctccttgagaggcagaggcaggtgaatttctgagtttgaggccagcctggtct
acaga ...... (Length, 10,546)

CCCCTGAGGAACAGCTCTCAGCTGGGAAGCATGGCGGACGGTCCTCATCTGCACACACTCCCAAAGAAGTT
TCTGAGGAATGGATCCTTGATTTTAAAAC ...... (Length, 2,017)

b

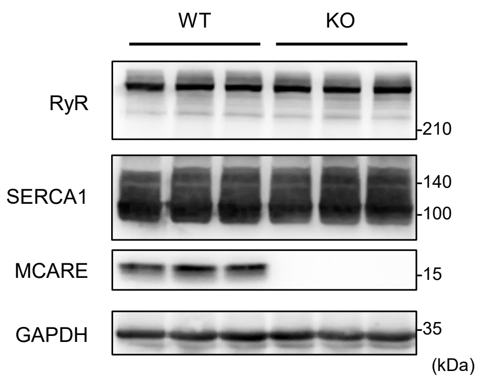

c

WT

```
 10         20         30         40         50         60
M  S  R  Q   P  E  P   Q  A  A   R  T  V  R   S  S  S   L  P  V
ATGAGTCGAC AGCCAGAGCC CCAGGCTGCA CGAACCGTGC GCTCCTCATC CCTCCCGGTG

        70         80         90        100        110        120
S  P  T  S   P  M  S   Q  Y  A   S  R  S  D   S  K  G   A  L  D
TCACCGACTT CGCCCATGTC TCAGTATGCT TCCCGCTCAG ACTCAAAAGG AGCTTTGGAC

       130        140        150        160        170        180
S  S  S  P   E  A  Y   T  E  D   D  K  T  E   E  D  I   P  A  P
AGCAGCAGCC CGGAAGCCTA TACAGAAGAT GACAAGACCG AGGAGGACAT ACCTGCCCCC

       190        200        210        220        230        240
S  N  Y  L   W  L  T   I  I  S   C  F  C  P   A  Y  P   V  N  I
AGTAACTATC TATGGCTCAC CATTATCTCG TGTTTCTGCC CAGCGTACCC GGTCAACATC

       250        260        270        280        290        300
V  A  L  V   F  S  I   M  S  L   N  S  Y  N   D  G  D   Y  E  G
GTGGCTTTGG TCTTCTCCAT CATGTCTCTG AACAGCTACA ATGATGGAGA CTACGAAGGA

       310        320        330        340        350        360
A  R  R  L   G  R  N   A  K  W   V  A  I  A   S  I  I   I  G  L
GCCAGGAGGC TGGGGCGGAA CGCCAAGTGG GTGGCCATTG CCTCCATCAT CATCGGCCTG

       370        380        390        400        410        420
V  I  I  G   V  S  C   A  V  H   F  S  R  N   P  *
GTCATCATCG GCGTCTCCTG TGCGGTGCAC TTCTCCAGGA ACCCCTGA
```

KO

```
 10         20         30         40         50         60
M  S  R  Q   P  E  P   Q  A  A   R  T  V  R   S  S  S   L  P  V
ATGAGTCGAC AGCCAGAGCC CCAGGCTGCA CGAACCGTGC GCTCCTCATC CCTCCCGGTG

        70         80         90        100        110        120
S  P  T  S   P  M  S   Q  Y  A   S  R  S  D   S  K  G   A  L  D
TCACCGACTT CGCCCATGTC TCAGTATGCT TCCCGCTCAG ACTCAAAAGG AGCTTTGGAC

       130        140        150        160        170        180
S  S  S  P   E  A  Y   T  E  D   D  K  T  E   E  D  I   P  A  P
AGCAGCAGCC CGGAAGCCTA TACAGAAGAT GACAAGACCG AGGAGGACAT ACCTGCCCCC

       190        200        210        220        230        240
S  N  Y  L   W  L  T   V  F  L   P  G  Q  H   R  G  F   G  L  L
AGTAACTATC TATGGCTCAC CGTGTTTCTA CCCGGTCAAC ATCGTGGCTT TGGTCTTCTC

       250        260        270        280        290        300
H  H  V  S   E  Q  L   Q  *  W   R  L  R  R   S  Q  E   A  G  A
CATCATGTCT CTGAACAGCT ACAATGATGG AGACTACGAA GGAGCCAGGA GGCTGGGGCG

       310        320        330        340        350        360
E  R  Q  V   G  G  H   C  L  H   H  H  R  P   G  H  H   R  R  L
GAACGCCAAG TGGGTGGCCA TTGCCTCCAT CATCATCGGC CTGGTCATCA TCGGCGTCTC

       370        380        390        400        410        420
L  C  G  A   L  L  Q   E  P  L
CTGTGCGGTG CACTTCTCCA GGAACCCCTG A
```

Supplementary Fig. 7 | Generation of *Mcare* KO mice. a, Genomic sequence of the *Mcare* gene with the sgRNA target sequence underlined in red. Exons and introns are highlighted in blue and orange, respectively. For the intron sequence and the third exon, only the first 100 bases are shown. **b**, Western blot of gastrocnemius muscle from WT and KO mice using RyR, SERCA1, MCARE, and GAPDH antibodies. **c**, Comparison of the amino acid and nucleotide sequences of MCARE in WT and *Mcare* KO mice.

a

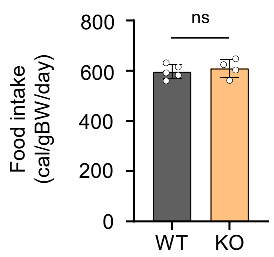

b

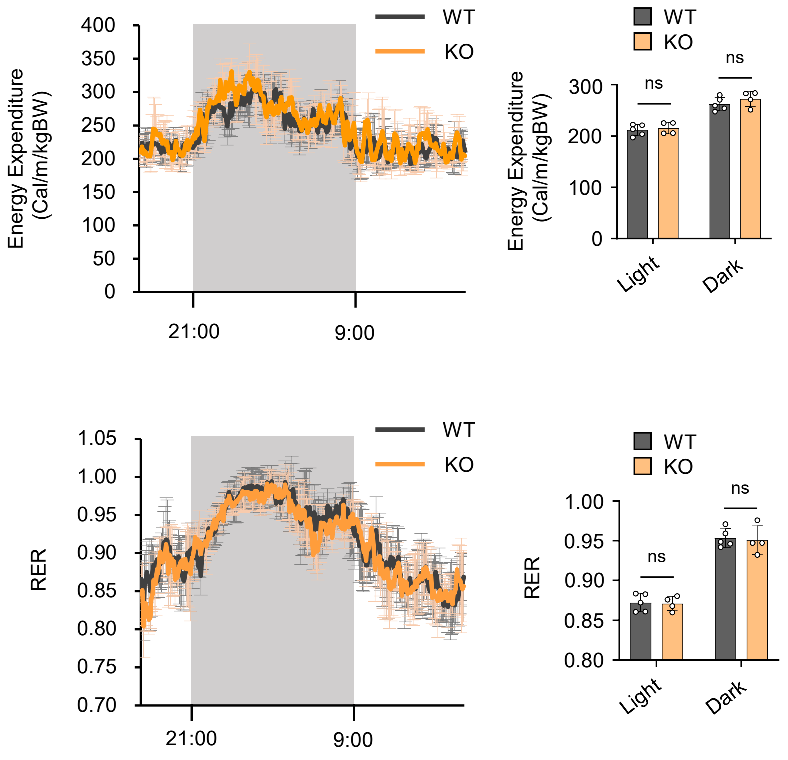

Supplementary Fig. 8 | *Mcare* deficiency does not affect energy metabolism in mice. a, Daily food intake of WT and KO littermates. **b**, Energy expenditure and RER were evaluated by O_2 consumption and CO_2 production over 24 h. The panels on the right show the average values for the light and dark phases. n = 5 (WT) and 4 (KO) mice. Error bars represent mean ± SD. Statistical significance was assessed using two-tailed unpaired Student's *t*-test.

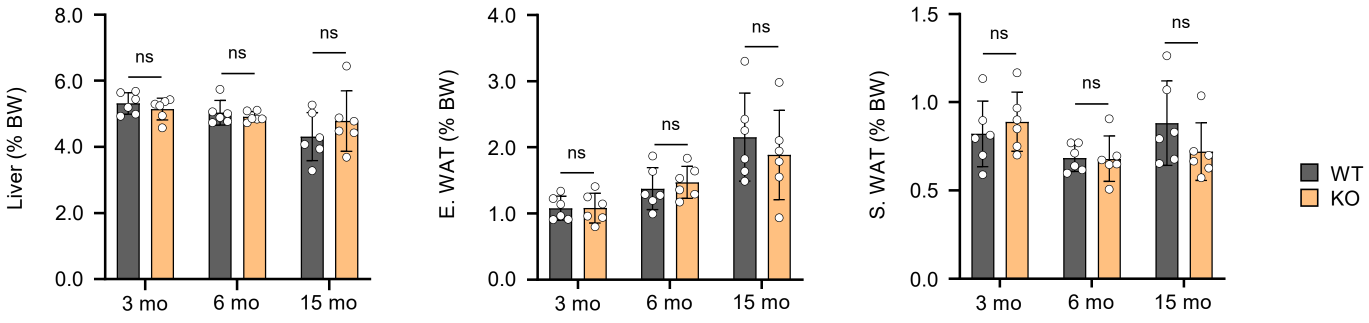

Supplementary Fig. 9 |*Mcare* KO does not affect liver or adipose tissue weight. The weights of the liver, epididymal white adipose tissue (E. WAT), and subcutaneous white adipose tissue (S. WAT) are shown as a percentage of body weight (%BW) in WT and *Mcare* KO mice at 3, 6, and 15 months of age. n = 6 mice. Error bars represent mean ± SD. Statistical significance was assessed using two-tailed unpaired Student's *t*-test.

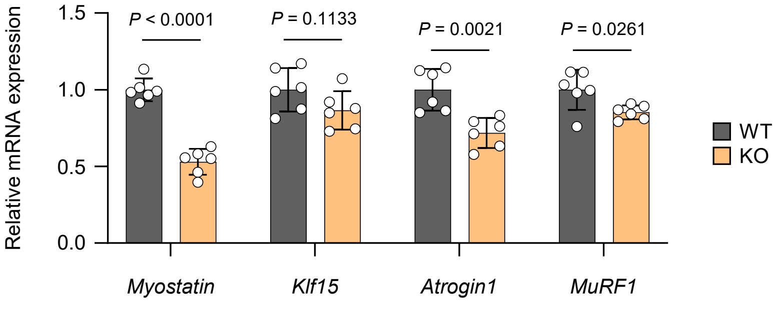

Supplementary Fig. 10 | Expression of muscle atrophy-related genes in *Mcare* KO mice. Relative mRNA expression of muscle atrophy-related genes in the quadriceps of WT and *Mcare* KO mice was measured by qRT-PCR. n = 6 mice. Error bars represent mean ± SD. Statistical significance was assessed using two-tailed unpaired Student's *t*-test.

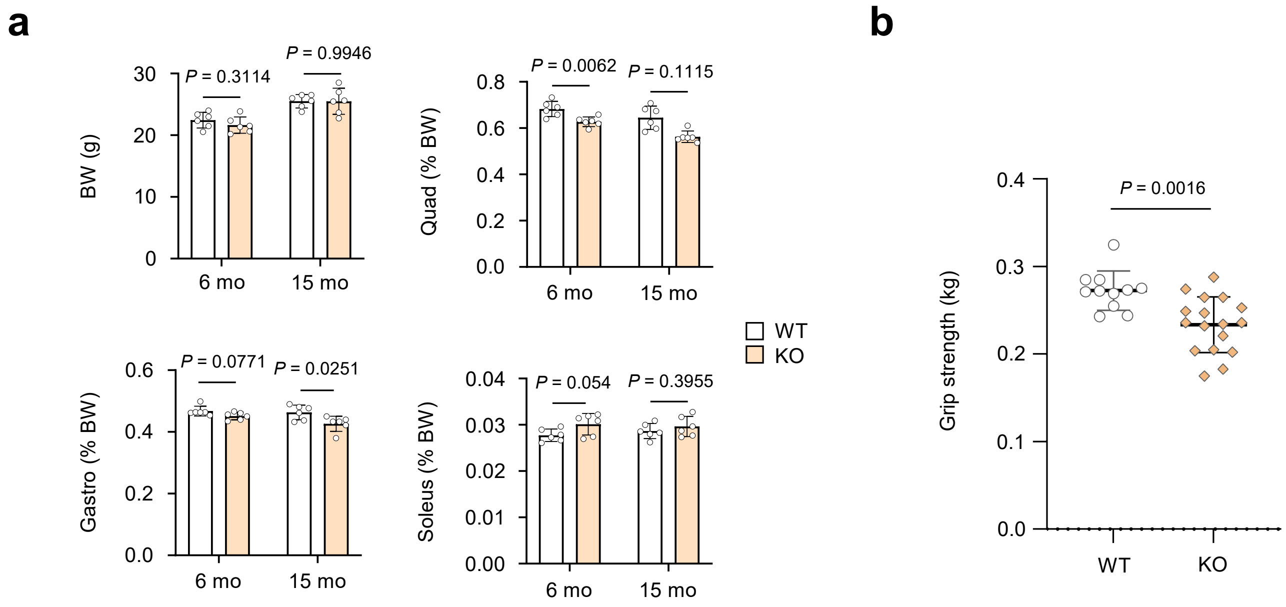

Supplementary Fig. 11 | *Mcare* deficiency causes muscle atrophy and weakness in female mice. a, Body weight (BW) and skeletal muscle weight expressed as a percentage of BW in female mice aged 6 and 15 months. n = 6 mice. **b**, Grip strength of 6-month-old female mice. n = 11 (WT) and 17 (KO) mice. Error bars represent mean ± SD. Statistical significance was assessed using two-tailed unpaired Student's *t*-test.

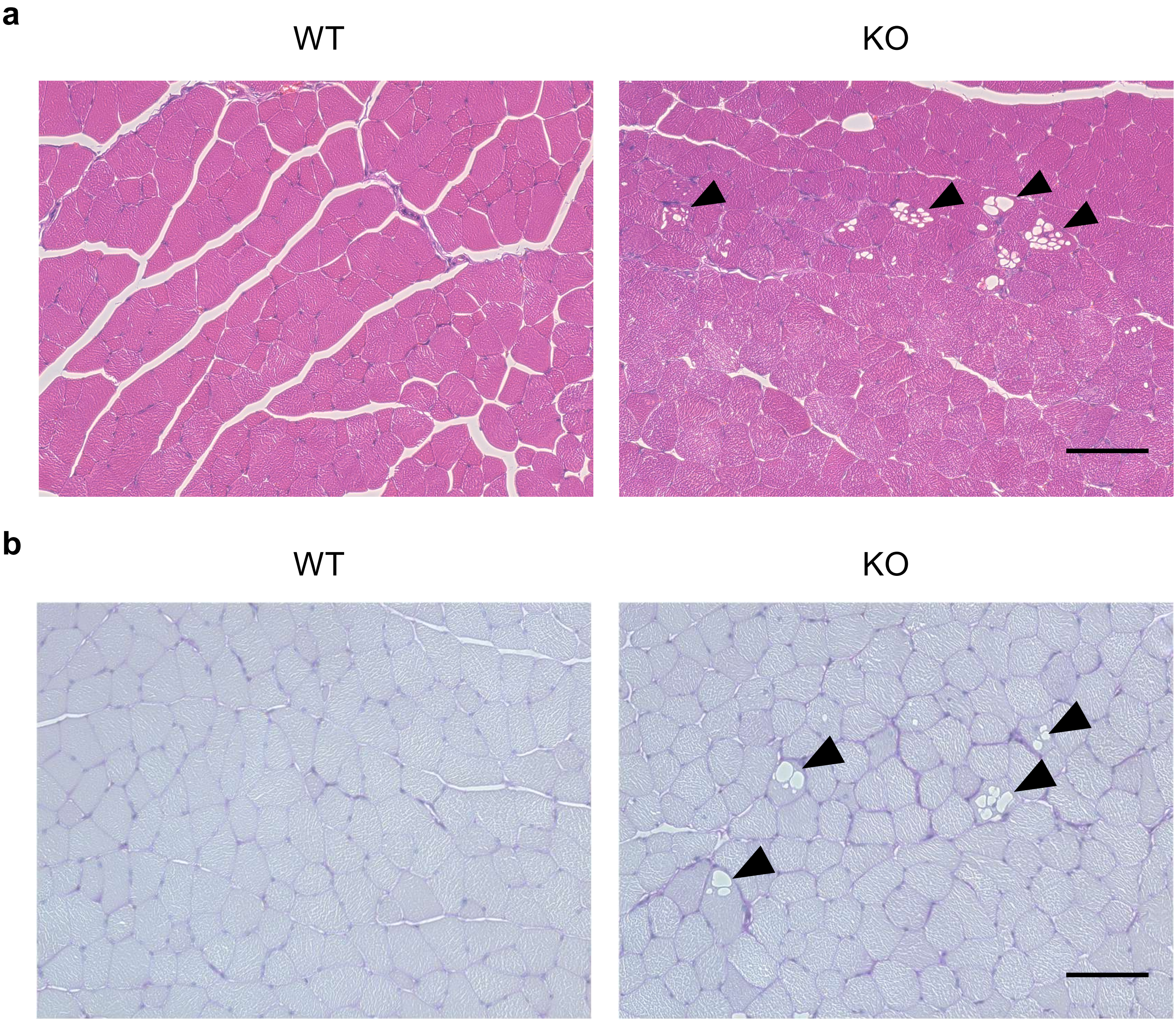

Supplementary Fig. 12 | Vacuolation in skeletal muscle fibers of KO mice. a, b, Quadriceps muscle sections from 6-month-old WT and KO mice stained with hematoxylin and eosin (HE; a) and periodic acid–Schiff (PAS; b). Arrowheads indicate representative vacuoles. Similar results were obtained from six independent mice. Scale bar, 100 µm.

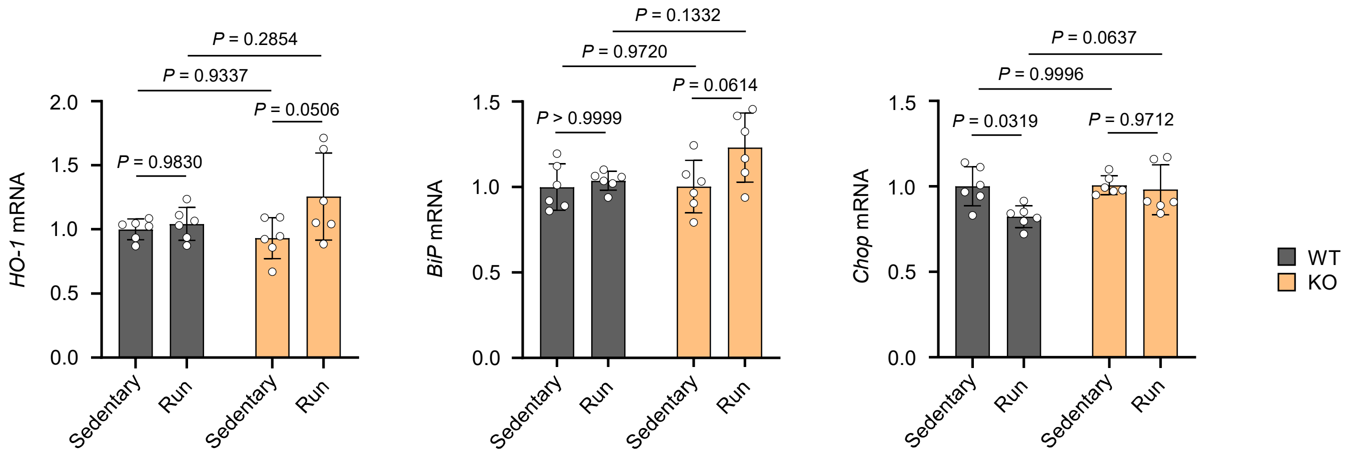

Supplementary Fig. 13 | *Atf3* upregulation in *Mcare* KO muscle following exercise is not accompanied by induction of canonical oxidative or ER stress markers. RT-PCR analysis of *HO-1*, *BiP*, and *Chop* mRNA levels in TA muscle of WT and *Mcare* KO mice under sedentary conditions and 60 min after a 30-min submaximal treadmill running protocol. n = 6 mice per group. Error bars represent mean ± SD. Statistical significance was assessed using two-way ANOVA with Tukey's multiple comparisons test.

Supplementary Table 1 | List of primer sequences used for quantitative RT-PCR analyses

Gene	Forward	Reverse
**18S**	ACCGCAGCTAGGAATAATGGA	GCCTCAGTTCCGAAAACCA
**Atf3**	GCTGCCAAGTGTCGAAACAAG	CAGTTTTCCAATGGCTTCAGG
**Atp2a1 (SERCA1)**	CACCACCAACCAGATGTCAG	TCAAGACCTCTCCCTCAGGA
**Atp2a2 (SERCA2)**	GTGAAGTGCCATCAGTATGACGG	GTGAGAGCAGTCTCGGTAGCTT
**Atp2a3 (SERCA3)**	TGCGGAAAGAGTTCACCCTGGA	GCGCTCAATTACACTCTCAGGAG
**Ddit3 (Chop)**	CTGCCTTTCACCTTGGAGAC	CGTTTCCTGGGGATGAGATA
**Fbxo32 (Atrogin1)**	GCAAACACTGCCACATTCTCTC	CTTGAGGGGAAAGTGAGACG
**HO-1**	GTGTCCAGAGAAGGCTTTAAGCT	TCTGCTTGTTGCGCTCTATCA
**Hspa5 (BiP)**	GAAAGGATGGTTAATGATGCTGAG	GTCTTCAATGTCCGCATCCTG
**Klf15**	ACCGAAATGCTCAGTGGGTTACCTA	GGAACAGAAGGCTTGCGAGTCA
**MCK**	GCTGTCCGTGGAAGCTCTCAACA	AATGAGCTGCTGCTGTTCCTG
**Mustn1**	GACATCAAGTCTAAGACATACC	GCTTCTCAAAGACTGTCTCG
**MyHC3**	TCCAAACCGTCTCTGCACTGTT	AGCGTACAAAGTGTGGGTGTGT
**Myogenin**	GCATGTAAGGTGTGTAAGAG	GCGCAGGATCTCCACTTTAG
**Myoregulin**	CAACGTTGCTAGGAGAACACC	GCTCTTGCCACTCATGTTCA
**Myostatin**	TGGCCATGATCTTGCTGTAACC	AGTCAAGCCCAAAGTCTCTC
**Nr4a3**	TCAGCCTTTTTGGAGCTGTT	TGAAGTCGATGCAGGACAAG
**Pax7**	CTGGATGAGGGCTCAGATGT	GGTTAGCTCCTGCCTGCTTA
**Ppargc1a(PGC-1α)**	TTCTGGGTGGATTGAAGTGGTG	TGTCAGTGCATCAAATGAGGGC
**Ptprc (CD45)**	TCACAAGCATGCATCCATCC	TTCCAAGAGATTGAACAAGGCA
**Tmem233 (Mcare)**	ACTTCGCCCATGTCTCAGTATGCTTC	GTCTCCATCATTGTAGCTGTTCAG
**Trim63 (MuRF1)**	ACCTGCTGGTGGAAAACATC	CTTCGTGTTCCTTGCACATC
