# Supplementary material for: MCARE enhances SERCA1 activity in fast-twitch muscle to maintain calcium handling and muscle integrity
Source: Nat Commun. 2025 Dec 10;17:629. doi: 10.1038/s41467-025-67358-4 (PMC12815910; doi:10.1038/s41467-025-67358-4)
Supplement: Supplementary file 2 — Description of Additional Supplementary Information [file 41467_2025_67358_MOESM2_ESM.pdf]

Description of Additional Supplementary Files

File Name: Supplementary Movie 1
Description: Representative movie showing EPSinduced transient Ca^{2+} fluorescence increases in control and MCARE-expressing C2C12 myotubes.

File Name: Supplementary Movie 2
Description: Representative movie showing EPSinduced contraction and relaxation of control and MCAREexpressing C2C12 myotubes.

File Name: Supplementary Movie 3
Description: Rippling muscle contraction following skin removal in *Mcare* KO mice compared with WT mice.

File Name: Supplementary Movie 4
Description: Rippling contractions in completely isolated hindlimb muscles from *Mcare* KO mice.
