# Supplementary material for: MCARE enhances SERCA1 activity in fast-twitch muscle to maintain calcium handling and muscle integrity
Source: Nat Commun. 2025 Dec 10;17:629. doi: 10.1038/s41467-025-67358-4 (PMC12815910; doi:10.1038/s41467-025-67358-4)
Supplement: Supplementary file 7 — Reporting Summary [file 41467_2025_67358_MOESM7_ESM.pdf]

nature portfolio

Corresponding author(s): Takashi Sasaki
Ryuichiro Sato

Last updated by author(s): Nov 7, 2025

Reporting Summary

Nature Portfolio wishes to improve the reproducibility of the work that we publish. This form provides structure for consistency and transparency in reporting. For further information on Nature Portfolio policies, see our Editorial Policies and the Editorial Policy Checklist.

Statistics

For all statistical analyses, confirm that the following items are present in the figure legend, table legend, main text, or Methods section.

n/a	Confirmed	
☐	☒	The exact sample size (n) for each experimental group/condition, given as a discrete number and unit of measurement
☐	☒	A statement on whether measurements were taken from distinct samples or whether the same sample was measured repeatedly
☐	☒	The statistical test(s) used AND whether they are one- or two-sided<br>*Only common tests should be described solely by name; describe more complex techniques in the Methods section.*
☐	☒	A description of all covariates tested
☐	☒	A description of any assumptions or corrections, such as tests of normality and adjustment for multiple comparisons
☐	☒	A full description of the statistical parameters including central tendency (e.g. means) or other basic estimates (e.g. regression coefficient) AND variation (e.g. standard deviation) or associated estimates of uncertainty (e.g. confidence intervals)
☐	☒	For null hypothesis testing, the test statistic (e.g. F, t, r) with confidence intervals, effect sizes, degrees of freedom and P value noted<br>*Give P values as exact values whenever suitable.*
☒	☐	For Bayesian analysis, information on the choice of priors and Markov chain Monte Carlo settings
☒	☐	For hierarchical and complex designs, identification of the appropriate level for tests and full reporting of outcomes
☐	☒	Estimates of effect sizes (e.g. Cohen's d, Pearson's r), indicating how they were calculated

Our web collection on statistics for biologists contains articles on many of the points above.

Software and code

Policy information about availability of computer code

Data collection	Western blot: Evolution-Capt Edge<br>qRT-PCR: StepOne Software (v2.3)<br>Microscopy: BZ-X800 Viewer or ZEN 2.6 (blue edition)
Data analysis	Graphpad Prism (v9.5.1)<br>SAS OnDemand for Academics<br>VW-9000 Motion Analyzer (1.0.0.2)<br>Fiji ImageJ (v2.14)<br>GENETYX (v16)

For manuscripts utilizing custom algorithms or software that are central to the research but not yet described in published literature, software must be made available to editors and reviewers. We strongly encourage code deposition in a community repository (e.g. GitHub). See the Nature Portfolio guidelines for submitting code & software for further information.

Data

Policy information about availability of data

All manuscripts must include a data availability statement. This statement should provide the following information, where applicable:

- Accession codes, unique identifiers, or web links for publicly available datasets
- A description of any restrictions on data availability
- For clinical datasets or third party data, please ensure that the statement adheres to our policy

All data supporting the findings of this study are available in the Source Data file. Source Data are provided with this paper.

Research involving human participants, their data, or biological material

Policy information about studies with human participants or human data. See also policy information about sex, gender (identity/presentation), and sexual orientation and race, ethnicity and racism.

Reporting on sex and gender	Study did not include human participants.
Reporting on race, ethnicity, or other socially relevant groupings	N/A
Population characteristics	N/A
Recruitment	N/A
Ethics oversight	N/A

Note that full information on the approval of the study protocol must also be provided in the manuscript.

Field-specific reporting

Please select the one below that is the best fit for your research. If you are not sure, read the appropriate sections before making your selection.

[x] Life sciences [ ] Behavioural & social sciences [ ] Ecological, evolutionary & environmental sciences

For a reference copy of the document with all sections, see nature.com/documents/nr-reporting-summary-flat.pdf

Life sciences study design

All studies must disclose on these points even when the disclosure is negative.

Sample size	No formal statistical methods were applied to determine the sample size; however, the chosen sample sizes align with those commonly used in similar studies and were sufficient to detect consistent, biologically meaningful differences. To ensure reproducibility, all experiments were conducted with at least three independent biological replicates.
Data exclusions	No data were excluded.
Replication	All reported experimental findings were reproducible across multiple biological replicates, demonstrating consistency in results. The exact numbers of biological replicates for each experiment are provided within the relevant figure legends.
Randomization	Age-matched mice with different genotypes were randomly assigned to study groups to minimize selection bias. Randomization was performed before the start of the experiment to ensure unbiased group allocation.
Blinding	Blinding was not performed for in vitro experiments because these assays were conducted by a single investigator using standardized and reproducible imaging and quantification procedures. For animal experiments, investigators were blinded to the genotype of the mice to prevent observer bias during data collection and analysis.

Reporting for specific materials, systems and methods

We require information from authors about some types of materials, experimental systems and methods used in many studies. Here, indicate whether each material, system or method listed is relevant to your study. If you are not sure if a list item applies to your research, read the appropriate section before selecting a response.

Materials & experimental systems

n/a	Involved in the study
	☒ Antibodies
	☒ Eukaryotic cell lines
☒	Palaeontology and archaeology
	☒ Animals and other organisms
☒	Clinical data
☒	Dual use research of concern
☒	Plants

Methods

n/a	Involved in the study
☒	ChIP-seq
☒	Flow cytometry
☒	MRI-based neuroimaging

Antibodies

Antibodies used

anti-MCARE antibody (Laboratory-generated)
anti-Caveolin-3 antibody (Santa Cruz Biotechnology, Cat. No. sc-5310)
anti-N-Cadherin antibody (Cell Signaling Technology, Cat. No. 4061)
anti-Lamin A/C antibody (Cell Signaling Technology, Cat. No. 2032)
anti-Tom20 antibody (Cell Signaling Technology, Cat. No. 42406)
anti-GM130 antibody (BD Bioscience, Cat. No. 610823)
anti-HSP90 antibody (Santa Cruz Biotechnology, Cat. No. sc-13119)
anti-RyR antibody (Santa Cruz Biotechnology, Cat. No. sc-376507)
anti-SERCA1 antibody (Cell Signaling Technology, Cat. No. 4219)
anti-SERCA2 antibody (Santa Cruz Biotechnology, Cat. No. sc-376235)
anti-FLAG M2 antibody (Sigma-Aldrich, Cat. No. F3165)
anti-Myc antibody (Sigma-Aldrich, Cat. No. C3956)
anti-GFP antibody (Cell Signaling Technology, Cat. No. 2956)
anti-GAPDH antibody (Proteintech. Cat. No. 10494-1-AP)
anti-CD31 antibody (Miltenyi Biotec, Cat. No. 130-119-662)
anti-CD140a antibody (Miltenyi Biotec, Cat. No. 130-101-905)
anti-CD11b MicroBeads (Miltenyi Biotec, Cat. No. 130-049-601)
anti-integrin-α7 antibody, Biotin (Miltenyi Biotec, Cat. No. 130-101-979)
Alexa Fluor 568 conjugated anti-rabbit IgG (Thermo Fisher Scientific, Cat. No. A-11011)
Alexa Fluor 488 conjugated anti-mouse IgG (Thermo Fisher Scientific, Cat. No. A-11001)

Validation

Commercially available antibodies are validated by the manufacturer (see technical data sheets accessable on the manufacturer's websites).

anti-Caveolin-3 antibody (Santa Cruz Biotechnology, Cat. No. sc-5310), https://www.scbt.com/p/caveolin-3-antibody-a-3?srsltid=AfmBOooBhwRWC9MEqOt7JOAGuAe9NVqhcjbaf-Xn-jEASL1hnyGhYQLW

anti-N-Cadherin antibody (Cell Signaling Technology, Cat. No. 4061), https://www.cellsignal.com/products/primary-antibodies/n-cadherin-antibody/4061?srsltid=AfmBOooqDHDvbJKWruMw_loDgIHZC5oBF0skH5when8B9kmAZ6SjSVYP

anti-Lamin A/C antibody (Cell Signaling Technology, Cat. No. 2032), https://www.cellsignal.com/products/primary-antibodies/lamin-a-c-antibody/2032?srsltid=AfmBOoprWa9pxV68FeK1yyIXEkbBz5UXXzdYO0FK0X9UbVtCRdpxyiJL

anti-Tom20 antibody (Cell Signaling Technology, Cat. No. 42406), https://www.cellsignal.com/products/primary-antibodies/tom20-d8t4n-rabbit-mab/42406?srsltid=AfmBOooxf4nN4UO5nVXiKigAEHpGRR5jSF93vZmrL70avqJ-q-qGBK53

anti-GM130 antibody (BD Bioscience, Cat. No. 610823), https://www.scbt.com/p/gm130-antibody-b-10?gad_source=1&gad_campaignid=1495372201&gclid=Cj0KCQjwoNzABhDbARIsALfY8VML237PhljC5KmEUydn2eQr9LOjRh0UhJ5XZcMCqRmASATWUi8MNtcaApefEALw_wcB

anti-HSP90 antibody (Santa Cruz Biotechnology, Cat. No. sc-13119), https://www.scbt.com/ja/p/hsp-90alpha-beta-antibody-f-8?srsltid=AfmBOoqTYuUQqfC2eWpY58CDf-cMDipih3ooEX26TUtpsdXVoUGB2w8c

anti-RyR antibody (Santa Cruz Biotechnology, Cat. No. sc-376507), https://www.scbt.com/p/ryr-antibody-f-1?srsltid=AfmBOopzMu35KVSFyxO5r1Fkj-WP1gPqJg1qDzxizCYdWhIGsgQWzrDo

anti-SERCA1 antibody (Cell Signaling Technology, Cat. No. 4219), https://www.cellsignal.jp/products/primary-antibodies/atp2a1-serca1-l24-antibody/4219

anti-SERCA2 antibody (Santa Cruz Biotechnology, Cat. No. sc-376235), https://www.scbt.com/p/serca2-antibody-f-1?srsltid=AfmBOooAj-hPkbbtsX8UyKV-cNY0Om6eUYMK1k27tus7hgLOXJwazd1O

anti-FLAG M2 antibody (Sigma-Aldrich, Cat. No. F3165), https://www.sigmaaldrich.com/JP/en/product/sigma/f3165?utm_source=google&utm_medium=cpc&utm_term=&utm_campaign=DSA_760&utm_content=google_cpc_DSA_DSA_760&gad_sou

rce=1&gclid=CjwKCAiAudG5BhAREiwAWMlSjA6ZJM-o3EtJFSSbvcR5l9gLl1o3HNlK1ddQ60P7ITJr7093gadLkRoCspEQAvD_BwE

anti-Myc antibody (Sigma-Aldrich, Cat. No. C3956), https://www.sigmaaldrich.com/JP/en/product/sigma/c3956?srsltid=AfmBOoo0ALVe9NAVW-ECt3zzjBb4bbkixkRp1XU7rRZWXy2HwUbxX1d4

anti-GFP antibody (Cell Signaling Technology, Cat. No. 2956), https://www.cellsignal.com/products/primary-antibodies/gfp-d5-1-rabbit-mab/2956?srsltid=AfmBOooUNXZXbU1laBlQYuQAQTegqFhCgmU0nkEFlhYbOyFKKpJhohYD

anti-GAPDH antibody (Proteintech. Cat. No. 10494-1-AP), https://www.ptglab.com/products/GAPDH-Antibody-10494-1-AP.htm

anti-CD31 antibody (Miltenyi Biotec, Cat. No. 130-119-662) , https://www.miltenyibiotec.com/JP-en/products/cd31-antibody-anti-mouse-390.html#conjugate=biotin:size=30-ug-in-200-ul

anti-CD140a antibody (Miltenyi Biotec, Cat. No. 130-101-905), https://www.miltenyibiotec.com/JP-en/products/cd140a-antibody-anti-mouse-apa5.html#conjugate=biotin:size=30-ug-in-1-ml

anti-CD11b MicroBeads (Miltenyi Biotec, Cat. No. 130-049-601), https://www.miltenyibiotec.com/UN-en/products/cd11b-microbeads-human-and-mouse.html#130-049-601

anti-integrin-α7 antibody, Biotin (Miltenyi Biotec, Cat. No. 130-101-979), https://www.miltenyibiotec.com/JP-en/products/integrin-a7-antibody-anti-mouse-3c12.html#conjugate=biotin:size=30-ug-in-200-ul

Alexa Fluor 568 conjugated anti-rabbit IgG (Thermo Fisher Scientific, Cat. No. A-11011), https://www.thermofisher.com/antibody/product/Goat-anti-Rabbit-IgG-H-L-Cross-Adsorbed-Secondary-Antibody-Polyclonal/A-11011

Alexa Fluor 488 conjugated anti-mouse IgG (Thermo Fisher Scientific, Cat. No. A-11001), https://www.thermofisher.com/antibody/product/Goat-anti-Mouse-IgG-H-L-Cross-Adsorbed-Secondary-Antibody-Polyclonal/A-11001

The MCARE antibody produced in our laboratory was validated using cultured cells overexpressing MCARE, as well as skeletal muscle tissue samples obtained from WT and Mcare KO mice.

Eukaryotic cell lines

Policy information about cell lines and Sex and Gender in Research

Cell line source(s)	C2C12 (Cat. No. CRL-1772) and HEK293 (Cat. No. CRL-1573) cells were purchased from American Type Culture Collection (ATCC).
Authentication	All cell lines were authenticated by evaluating their growth conditions, morphology, and specific gene expression profiles.
Mycoplasma contamination	All cell lines were regularly tested for mycoplasma contamination and confirmed to be negative.
Commonly misidentified lines (See ICLAC register)	No commonly misidentified lines were used in this work.

Animals and other research organisms

Policy information about studies involving animals; ARRIVE guidelines recommended for reporting animal research, and Sex and Gender in Research

Laboratory animals	Species: Mus musculus<br>Strain and background: All animals were on a C57BL/6J background.<br>Source: Wild-type C57BL/6J mice were purchased from CLEA Japan, Inc. for experiments using only wild-type animals. Mcare KO mice and their wild-type littermates were bred and maintained at the University of Tokyo.<br>Age: Mice ranging from 3 weeks to 15 months of age were used. The exact ages used in each experiment are specified in the corresponding figure legends or Methods section.
Wild animals	This study did not involve wild animals.
Reporting on sex	Both male and female mice were used in this study; however, most experiments were performed using male mice to minimize variability due to hormonal cycles. Selected experiments (e.g., muscle weight and grip strength measurements) were also conducted in female mice, which showed comparable results to those of males.
Field-collected samples	This study did not involve sample collection in the field.
Ethics oversight	All animal experiments were conducted in accordance with the relevant ethical regulations and were approved by the Animal Usage Committee of the University of Tokyo under protocol numbers P19-016 and P22-125.

Note that full information on the approval of the study protocol must also be provided in the manuscript.

Plants

Seed stocks

Report on the source of all seed stocks or other plant material used. If applicable, state the seed stock centre and catalogue number. If plant specimens were collected from the field, describe the collection location, date and sampling procedures.

Novel plant genotypes

Describe the methods by which all novel plant genotypes were produced. This includes those generated by transgenic approaches, gene editing, chemical/radiation-based mutagenesis and hybridization. For transgenic lines, describe the transformation method, the number of independent lines analyzed and the generation upon which experiments were performed. For gene-edited lines, describe the editor used, the endogenous sequence targeted for editing, the targeting guide RNA sequence (if applicable) and how the editor was applied.

Authentication

Describe any authentication procedures for each seed stock used or novel genotype generated. Describe any experiments used to assess the effect of a mutation and, where applicable, how potential secondary effects (e.g. second site T-DNA insertions, mosiacism, off-target gene editing) were examined.
